# Sentiment Analysis and Emotion Understanding during the COVID-19 Pandemic in Spain and Its Impact on Digital Ecosystems

**DOI:** 10.3390/ijerph17155542

**Published:** 2020-07-31

**Authors:** Carlos de las Heras-Pedrosa, Pablo Sánchez-Núñez, José Ignacio Peláez

**Affiliations:** 1 Department of Audiovisual Communication and Advertising, Faculty of Communication Sciences, Universidad de Málaga, 29071 Malaga, Spain; 2 Joint-PhD Programme in Communication, Universidad de Málaga, 29071 Malaga, Spain; psancheznunez@uma.es; 3 Department of Languages and Computer Science, Higher Technical School of Computer Engineering, Universidad de Málaga, 29071 Malaga, Spain; jipelaez@uma.es

**Keywords:** coronavirus, COVID-19, SARS-CoV-2, public health, health communication, sentiment analysis, emotion understanding, opinion mining, risk communication, social media

## Abstract

COVID-19 has changed our lives forever. The world we knew until now has been transformed and nowadays we live in a completely new scenario in a perpetual restructuring transition, in which the way we live, relate, and communicate with others has been altered permanently. Within this context, risk communication is playing a decisive role when informing, transmitting, and channeling the flow of information in society. COVID-19 has posed a real pandemic risk management challenge in terms of impact, preparedness, response, and mitigation by governments, health organizations, non-governmental organizations (NGOs), mass media, and stakeholders. In this study, we monitored the digital ecosystems during March and April 2020, and we obtained a sample of 106,261 communications through the analysis of APIs and Web Scraping techniques. This study examines how social media has affected risk communication in uncertain contexts and its impact on the emotions and sentiments derived from the semantic analysis in Spanish society during the COVID-19 pandemic.

## 1. Introduction

The outbreak of the coronavirus disease (COVID-19) was first reported by the Wuhan Municipal Health and Safety Commission (Hubei Province, China) on 31 December 2019. One month later, the Emergency Committee of the International Health Regulations [1] declared the new coronavirus outbreak as a Public Health Emergency of International Importance (PHEI) at its meeting on 30 January 2020 [2].

Five months after the official announcement, the virus has infected more than 6,193,548 people worldwide and killed around 372,479 people [3], bringing catastrophic consequences for society [4], completely collapsing health systems in different countries [5] and generating a strong economic recession worldwide [6].

Throughout the history of mankind, societies have been faced with crises of various kinds and of very diverse natures, such as civil conflicts, financial crises, crises caused by the management and export of energy resources or emergencies caused by the impact of diseases and epidemics, among others [7]. It is only necessary to recall some of the events of the past 20th century to be able to appreciate how crises have conditioned our existence and have modified our way of living and understanding the world around us [8,9].

However, the current crisis caused by COVID-19 is developing in a social–technological scenario that is unprecedented for society. We are in a highly globalized world, where migration flexibility, free transit between countries, and the development and use of Information and Communication Technologies (ICTs) are fully established [10,11]. In addition, we must highlight the growing interconnection between the world’s economies, which is reflected in the flow of goods, capital, people, and ideas [12]. These facts and others associated with contemporary societies are making us face an old enemy of humanity as they are in this case, viruses such as the COVID-19 pandemic in a completely different context, and it is in this new scenario where areas such as communication, and in particular crisis communication, have become essential to manage and combat crises [8,13].

Crisis communication is fundamental in risk situations since it is responsible for maintaining an accessible and transparent relationship in the different flows and transmissions of information and communication, which is the premise of collective action. However, crisis communication is not only about transmitting knowledge but also about finding ways of transmitting comprehensive information that reflects uncertainty and enables the public to make fact-based decisions about our case, about health [14,15,16].

The population does not always understand the complex professional knowledge about risk, so crisis communication through its professionals must facilitate the understanding of such knowledge in content in a simple and explicit form so that it can be easily understood by all citizens, regardless of their social, cultural or educational level. Society needs to feel informed by qualified experts; otherwise, they will seek out the information, which may be unproven, rumored, or simply false news. Likewise, risk communication must maintain a balance between being neither too centralized nor too decentralized and allow the permeability of information between the different layers of the information process, and all with the ultimate goal of being identified by the population as a trusted source of information [17,18]. In this process, the different stakeholders must fulfill their responsibility according to their roles and keep the communication network functioning. 

Without a doubt, crisis communication is fundamental when it comes to transmitting security, calmness, and credibility. It oversees forging emotions that generate value to better face the different crises that occur. If this is not the case, it produces an effect of mistrust and lack of credibility in the population, directly affecting the emotions of the target audience. For Covello et al. (2001) [19], risks aroused by feelings of sadness, fear, anger, or mistrust will be perceived as greater than the risks that are not. For government institutions or agencies that lack credibility and trust, such feelings will increase in the face of trusted agencies or institutions. In this line, the public health risks associated with pandemics, which produce deaths or have irreversible consequences in a country, will generate a greater feeling of lack of protection and loss of trust among citizens [10,18,19].

One way of estimating, measuring or assessing crisis communication would be through the study of the emotions and feelings expressed by the public, and to do so without being questioned, to avoid what Arrow [20] calls the “strategic aspect of decisions”, where the person being questioned adapts his or her response to his or her interests. It is in this context that social media becomes essential, as it is a virtual space where society usually expresses itself freely (they are not asked), becoming an essential source from which to gather such information. 

The aim of this research work is to analyze the crisis communication in the field of public health that has been carried out during the COVID-19 pandemic, by measuring the emotions that have been generated in the population, and the crisis communication strategy that has been carried out by the Spanish Government. 

The example of Spain was selected as a case study. Under Royal Decree 463/2020 of 14 March Spain declared a state of alarm for the management of the health crisis caused by COVID-19 [21], positioning itself as one of the countries in the world most affected by the pandemic, with the highest number of cases per million inhabitants, adding up to a total of 239,638 confirmed cases of COVID-19 by PCR and the second-highest number of deaths according to population, with a total of 27,127 deaths [22]. 

**Figure 1:**
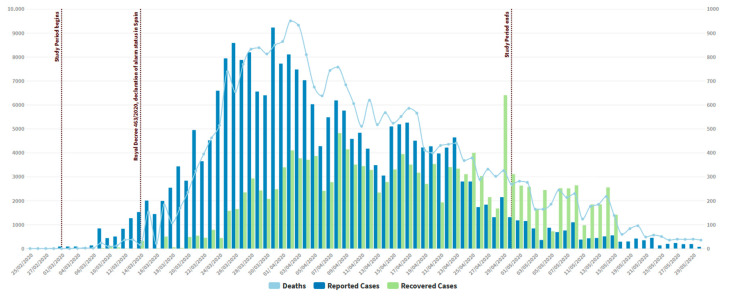
The daily evolution of reported cases, deaths, and recovered cases from the COVID-19 pandemic in Spain. Source: Ministry of Health of the Government of Spain [23].

Figure 1 shows the trend in reported cases of COVID-19, deaths, and recoveries patients between 25 February 2020 and 29 May 2020. It is verified that the highest level of cases by coronavirus was on 31 March 2020 and deceased people on April 1. After these days, the curve of new infections and deaths trended downward and recovered people were increasing. 

**Figure 2:**
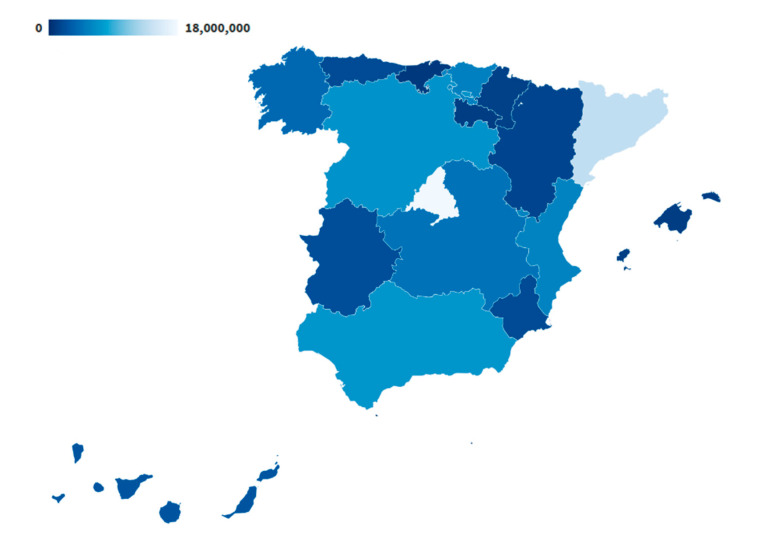
Masks that the Spanish Government has distributed to each autonomous community from March 10 to May 29 [24].

Face masks were a problem, especially at the beginning of the pandemic. Initially, they were not available to the general population, so they were only used for those most at risk of infection, such as health care, security forces, and the military. The Government of Spain was responsible for the purchase and distribution of face masks to the autonomous communities. Figure 2 shows the distribution of face masks carried out by the government between 10 March and 29 May. Madrid and Catalonia, with the highest number of infections from COVID-19, were where most face masks were distributed.

This research work represents a pioneering challenge in the field of risk communication research. During the months of March and April 2020, various digital ecosystems were analyzed, and a sample of 106,261 communications was obtained through the analysis of APIs and Web Scraping techniques. The study analyzes, through social media, how risk communication management has been affected during the COVID-19 pandemic, led by the main governing bodies, health organizations, and the collaboration of the main stakeholders (media). Moreover, the study investigates how COVID-19 has impacted society and the various effects on the emotions and feelings (anger, disgust, sadness, and fear) of the population [25]. 

Key variables in risk communication theories and the risk perception model are identified and were introduced into the framework of strategic health risk communication, to assess how the risk content of COVID-19 was or was not communicated to the population. 

The work is organized as follows: in Section 2, the materials and methods are presented; in Section 3, the results are obtained; and in Section 4, the discussion and conclusions finish the work with future research lines.

## 2. Materials and Methods 

This research collected data directly in real time from the main media and digital ecosystems: Twitter, YouTube, Instagram, official press websites, and internet forums. The data collection started on March 1 and finished on 30 April 2020. A sample of 106,261 communications was obtained. These data were comprised of written communications related to the COVID-19 pandemic in Spain. The collected communications were anonymized, eliminating authorship data, location, source, images, hyperlinks, and non-textual components, to leave only the text corpus.

The study universe comprised thirty-two million Spanish citizens, accounting for roughly 68% of the Spanish population that are considered as social media and internet users, according to the National Institute of Statistics of Spain (INE) data (forecast on 1 July 2019) [26]. Each internet user in Spain had the same odds of being included in this research. 

On the one hand, the inclusion criteria were:

- The communication makes explicit reference to the COVID-19 pandemic in Spain.
- The communication is public and can be viewed without the need for a subscription to the data source or explicit permission from the sender of the communication.
- The author’s reported age, when available, was over 18 years old as of the start of the end of the study (30 April 2020).
- The communication is written in Spanish.
- On the other hand, the exclusion criteria were:
- The communication does not come from an advertising campaign.
- The communication has not been generated by automatic procedural methods (bots, fake posts, among others).

One of the most usual problems that we must deal with when using information from digital ecosystems is detecting spammers, fake information generated by bots, which tries to influence or modify the perceived opinion on existing information. To detect and discard this type of information we have implemented different types of algorithms based on Support Vector Machine (SVM) techniques which can detect the patterns of this kind of communications, such as the age of the account (in days), the number of comments from the account, follower/following ratio, and the ratio of messages containing URLs. To prevent the effect of spammers, in this work we implemented and applied filters previously defined and tested in other scientific works [27,28].

The emotion information from each communication was extracted employing the natural language analysis tools provided by the IBM Watson Analytics service [29]. The emotional intensity was measured in a 0 to 1 scale, where 0 represents the complete absence of this emotion; and 1 represents an absolute high intensity of the emotion. In total, this study measured the emotional intensity of four primal emotions—anger, fear, disgust, and sadness.

To detect and measure the primary emotions in this study we used the services provided by the IBM Watson system. Watson is a cognitive computing platform that combines a DeepQA architecture, with AI algorithms and Big Data to solve questions in the domain of natural language. This platform offers a wide range of services including Discovery, Knowledge Studio, Language Translator, Natural Language Classifier/Understanding, and Personality Insights among others.

Watson has an overall precision of 97% in natural language processing and has been widely compared with other systems, as well as with humans, and in both cases, it has obtained very satisfactory results. For this reason, this system has been widely used in different scientific works where it has further proved its capabilities on Natural Language Processing (NLP) tasks [30,31,32,33,34,35].

In this work, we made use of the Natural Language Understanding service from the IBM Watson platform which, given an input text, provides an analysis of syntactic characteristics as well as information on categories, concepts, emotions, entities, keywords, metadata, relationships, and semantic roles.

The reliability of the resultant emotion information was tested using the Interval Majority Aggregation Operator (ISMA-OWA) [36], which is designed for Decision Making in Social Media with Consistent Data, leveraged by the combination of computational intelligence and Big Data techniques [37]. 

To obtain representative results, when analyzing with information extracted from digital ecosystems, it is important to ensure a correct representation of such information and its quality. 

When people express opinions in communications, they do not do so in numerical value with a fixed scale, they use natural language expressions such as “this is great” or “this is not so good”, so we employed the intervalar representation proposed in [36,38] instead of a numerical scale. The main advantage of this approach is that intervals represent the information within communication in a way that is more similar to the way people express themselves in digital ecosystems, thus reducing the loss of information associated with forcing linguistic data to a hard-numerical scale.

Furthermore, regarding information quality, an important aspect that we must consider when assessing the validity of this information is to ensure that such information has been expressed with knowledge of the topic at hand and not at random. Another advantage of the usage of an intervalar representation of digital ecosystem data is the availability of consistency indices that can be applied to the matrices obtained from communications to detect inconsistencies derived from uninformed opinions. For this purpose, in this work, we employed the CI+ index defined in [39].

The frequency of the words comprising the sample of communications was calculated using a natural language processing algorithm implemented in Python 3, using the Natural Language Toolkit (NLTK) [40]. Moreover, the emotion polarity (positive or negative) was measured using a multilayer perceptron model, trained to classify the emotional weight of written communications [38,41]. 

The Python NLTK library is an open-source programming library for working with natural language data which incorporate functions that allow for the determination of the frequency of words in a text while discarding stop-words, that is words that are very common to a language but do not convey any significant information such as “the”, “a” and “very”. Furthermore, the NLTK library serves as a pre-processing tool to use other Artificial Intelligence tools such as Artificial Neural Networks such as the multi-layered perceptron that we used in this work to detect the polarity of communications.

A multi-layered perceptron (MLP) is a widely used artificial neural network architecture that utilizes a technique known as supervised training to learn how to differentiate data that is not linearly separable. In this case, we trained our MPL with a set of communications created by the Spanish Society for Natural Language Processing (SEPLN) which contains over 100,000 natural language texts tagged with the polarity of each communication, that is, each communication contained metadata that indicated if the message was positive or negative. There are other techniques for Natural Language Sentiment Analysis, such as Naïve Bayes, or Support Vector Machines, but we opted for the MLP approach since it can learn complex relationships and it does not enforce any sort of constraint concerning the input data [42].

To further improve the qualitative analysis, the above-mentioned information regarding the volume of communications, the frequency of words and the emotion expressed by each communication was contrasted to determine the information pathways between mass media, government, political parties, employers’ confederation, non-governmental organizations (NGOs), trade unions, the World Health Organization (WHO), among others. This approach provides a graphical representation of the information fluxes about the COVID-19 disease in Spain. 

For the analysis of the messages emitted by the Spanish government, a content analysis of all press releases during the period of study was carried out. Messages were classified as positive, neutral, or negative by selecting the most significant words from them. The frequency of repetition of these words was another objective of this content analysis. The result has been shown through a word cloud representative of the emotions and feelings expressed by the government in its press releases.

The analysis of content permits inferences to be reproduced based on specific characteristics identified in the messages [43,44]. This type of analysis allows for the discovery of tendencies and the revelation of differences in content communication. Likewise, this allows the comparison of messages and means of communication, and the identification of intentions, appeals. To this effect, value and frequency analysis were used [45].

## 3. Results

### 3.1. Communication Structure with Stakeholders

Since the beginning of the pandemic, the structure organized by the government has involved relations between the Spanish government (the Health Alert Coordination Center, which is part of the Ministry of Health) and the governments of the autonomous communities, the National Epidemiology Center, the National Microbiology Center and the international organization’s World Health Organization (WHO), the European Disease Control Center and the European Commission [46].

To raise awareness and inform public opinion, the Spanish government designed a communication strategy articulated in four actions that had the use of the mass media as channels of transmission of COVID- 19 information as a main objective:

(a) Weekly appearances of the President of the Government.
(b) Daily press conferences chaired jointly by the following ministers: Minister of Health, who is responsible for the state of alarm decreed in the country; Minister of Defense, who is responsible for the military forces; Minister of the Interior, who is responsible for the State security forces and Minister of Transport. All of them were accompanied by experts in each of the areas. The ministers sent out a political message and the experts went into detail about the actions being taken. With a press conference format, online questions from the main Spanish and foreign media were admitted. However, this format underwent the first modification after the second week being responsible for the press conferences the so-called “Technical Committee for monitoring the coronavirus pandemic in Spain” consisting only of experts of the different ministries. On 25 April, there was a new restructuring of the press conferences, leaving only the director of the Health Alert and Emergency Coordination Centre of the Ministry of Health as the health expert. This last change is censored by the communications media.
(c) Press release. After the appearance at a press conference, the communication department of the Ministry of Health sent a press release to all the media.
(d) Interviews with ministers. Another of the government’s actions was to make its Cabinet available to the media for interviews.

To reinforce the previous actions, on March 15, the state government launched the advertising campaign *#*EsteVirusLoParamosUnidos. This campaign is adapted for television, press, radio, outdoor advertising, and social networks.

**Figure 3:**
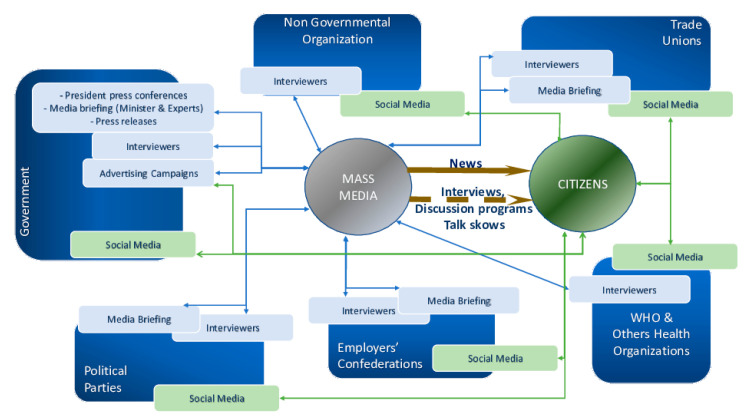
Interrelations between the main stakeholders and the citizens.

Figure 3 shows the main communication flows established in the government’s strategy. Except for social networks and outdoor advertising that are directly focused on citizens, the rest of the information is conducted through the media.

In Spain, the decreed state of alarm requires the total confinement of the population. Royal Decree-Law 10/2020, of 29 March 2020 [47], establishes the minimum essential services of first necessity such as all those necessary for the supply of food to the population. The minimum distance was made to be one and a half meters. Except for these cases, the rest of the population must carry out their work by teleworking, and if this work is not possible, the government approved Royal Decree-Law 8/2020 of 17 March on extraordinary urgent measures to cope with the economic and social impact of COVID-19 [48], which regulates emergency procedures to combat the economic and social impact of the pandemic, denominated as the Temporary Employment Regulation File (TERF). The number of workers affected by the TERF was two million on 3 April [49]. The high number of TERF requests blocks the administration from responding to the citizens with a decrease of the collection of these aids and the decapitalization of these workers in some cases without the possibility of paying the rents of their houses or simply buying the necessary food for the family. Non-Governmental Organizations and food banks have a crucial role to supply the neediest in the population. During confinement, the media are not left out. Their workers follow their work from their homes. On televisions, these measures cause programs to be suspended and replaced by new programming offering coronavirus specials. These programs have a structure of news, interviews with experts or politicians, discussion programs or talk shows where COVID-19 and the situation that citizens are experiencing are analyzed.

Due to the uncertainty of the situation and the isolation in their homes, citizens are consuming more television. Thus, the month of March and later April became the months with the highest television audience in Spanish history. March data show an average consumption of 282 min per person per day (4 h and 42 min). The average number of people who had watched TV for at least one minute a day was 369 min (6 h and 9 min) [50]. The progression in the television audience continued in the month of April with numbers never seen in the conventional Spanish television with 302 min (5 h and 2 min) and 395 min (6 h and 35 min) respectively. In addition to television coverage, 33.6 million Spaniards consumed this medium daily, representing 74.2% of the population [51].

The serious effects on the economy caused by the crisis determine that new actors acquire an active role in communication by modifying the initial panorama organized by the government. Political parties, the Confederation of Employers and trade unions are configured as sources of information. These new stakeholders also offer interviews to the communication media, organize press conferences, and finally communicate with citizens directly through social media (see Figure 1).

Therefore, the stakeholder structure created by the government is increased by other social actors who have their own opinion on the management of the pandemic. All of them have in common the use of the media to convey their messages to the citizens, converting these media as the main interlocutors with the population. The high consumption of television makes it the main means of information used by citizens.

Public and private televisions in Spain broadcast the press conferences of the different stakeholders and the appearances of the President of the Government. This is referred to in Figure 1 as “news”.

The different ideological tendencies of the television channels in Spain mean that their interview programs with experts and television debates do not follow a single argument in support of the government’s management. These messages feature contradictory opinions that the media convey to the public as interviews, discussion programs, and talk shows, which increase uncertainty among citizens (Figure 3).

### 3.2. Comparison between the Tone of the Messages Sent by the Government and the Feelings and Emotions Generated in the Population

In a public health crisis like the one Spain is experiencing, a transparent and empathetic communication style would generate citizen confidence and would be more effective if politicians and experts unanimously tried to stimulate the population to take a positive stance towards the pandemic and the health and economic alert measures imposed by the government. Although the generation of trust must be essential in a crisis, the analysis carried out shows the public’s distrust of scientific experts and government representatives for a variety of reasons such as access to conflicting sources of information, contradictions in scientific reasoning, changes in decision-making and, above all, political confrontations.

Trust and credibility, demonstrated through empathy, experience, honesty, and transparency, are essential elements of public health crisis communication [18].

**Figure 4:**
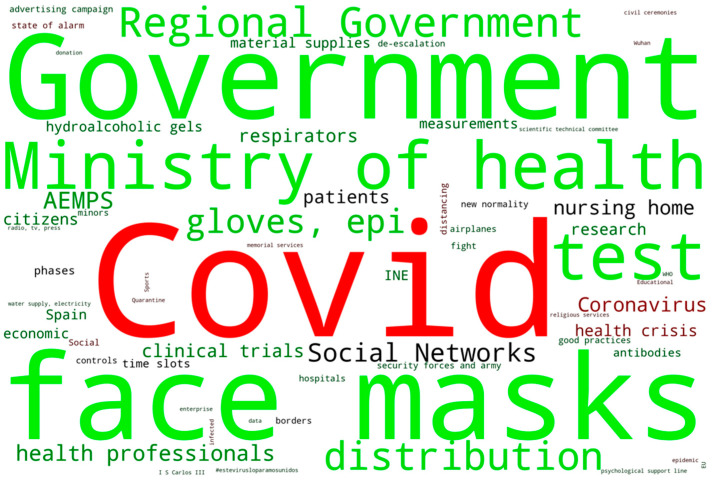
Word cloud generated from the press releases of the Ministry of Health during the period 1 March 2020 until 30 April 2020 related to terms about COVID-19.

Figure 4 analyzes the messages transmitted by the Ministry of Health in its press releases between March and April. In green, the positive messages were determined, in black the neutral ones and in red the negative ones. The word size indicates the frequency of repetition in the press releases. As can be seen in the word cloud, the negative word “COVID” is the most used by the government in its communications. This is followed at a distance by “coronavirus” and “health crisis” with a dark red color that indicates their use in negative messages, but also in neutral tones. “Social Networks” is a neutral term used mainly to explain the social network campaigns implemented by the government. It is followed by “patients” and “nursing home”. However, the most remarkable thing about this word cloud is its words in the green. The communication made by the communication office of the Ministry of Health has always wanted to give a positive view in all their messages, with “Government” as the most used word, followed by “face masks”, “Ministry of Health” or “test”. This could indicate a lack of transparency about the situation the country was going through. None of these press releases refer to either the infected or the dead. Attempts are made to give a protagonist role, at times, to all the actions carried out by the government.

**Figure 5:**
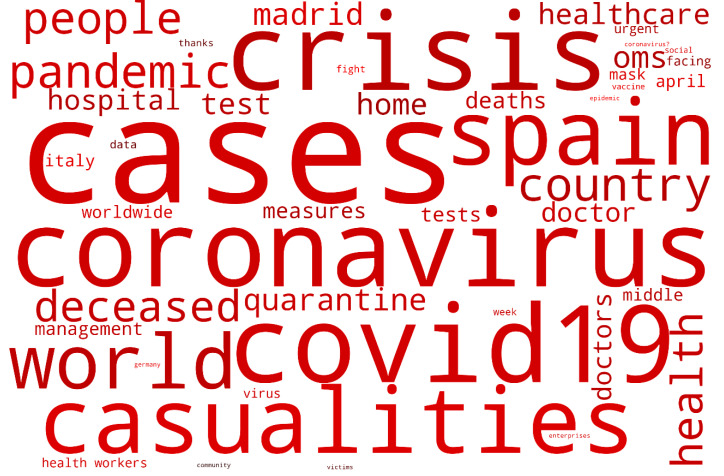
Word cloud generated during the period 1 March 2020 until 30 April 2020 related to terms about COVID-19.

In contrast, Figure 5 shows the results of the 106,261 listings made on social media between the same months and shows the feelings and emotions of the population. On this occasion, the word “cases” is the most representative that reflects the number of infections suffered in the country. It is followed by the word “crisis”, which represents the public health crisis but also the economic one. The terms “COVID” and “Coronavirus” are strongly represented, as well as “Spain” and “world” which represent the concern of the population in the face of a pandemic of this magnitude. “Casualties” is another of the most significant words and is indicative of all those people who have benefited from the TERF and who have not yet received the promised aid from the government. The positive messages sent by the government and its experts are counterbalanced by the volume of opinion generated by the media and especially the generalist televisions.

Some reasons include political parties’ criticism of the government’s management, contradictions of the experts, the constant increase of infected and dead, Spain being among the most affected countries, the state of confinement suffered by society not always in the best conditions, the anxiety of not having financial resources, the population’s insecurity in the face of a public health crisis with global effects that are caused by millions of infected people and hundreds of thousands of deaths in the world.

All these reasons generate negative feelings and emotions, causing uncertainty and fear among citizens. Digital ecosystems reflect this trend in a word cloud with a markedly negative character (Figure 5).

### 3.3. Communications that Generate the Greatest Emotional and Sentimental Impact on Society during the COVID-19 Pandemic

The communications that have the greatest impact on four of the main emotions of the population—fear, sadness, disgust, and anger—are presented. The study has allowed for the determination of the reaction of the population concerning the COVID-19 pandemic and the crisis communication carried out by the government, determining the themes and the feelings of the communications associated with the crisis communication.

To this end, the emotion graph corresponding to the period of study is first determined, determining the peaks of emotion that are significant, and those news patterns that generate greater presence and reach in digital ecosystems. Secondly, the topics that have most influenced these emotions are analyzed and the patterns that generate them are concluded.

#### 3.3.1. Association of Disgust Communications Connected with Management and Its Emotional Impact during COVID-19 Pandemic

**Figure 6:**
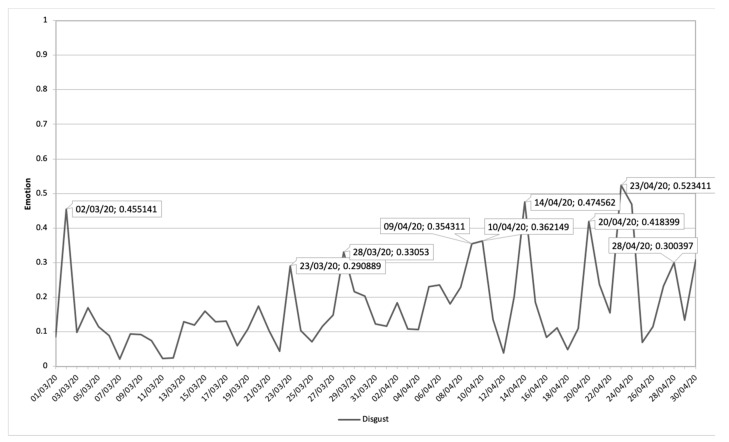
Disgust emotion during the period 1March 2020 until 30 April 2020 related to terms about COVID-19.

Figure 6 shows the evolution of the Disgust emotion during the study period, where nine peaks can be distinguished where the emotion shows a significant increase.

In Table 1, the communications that had the greatest impact on this increase are analyzed in chronological order from 1 March 2020 to 30 April 2020. From these communications, the management of the pandemic is the general theme that most impacts the emotion treated. Aspects such as: blaming the pandemic on groups that can be grouped by religion, sex, use of the security forces to censor the population’s opinion; lack of care for weak sectors such as the elderly; and the purchase of health material are the conversations that predominate in digital ecosystems.

**Figure 7:**
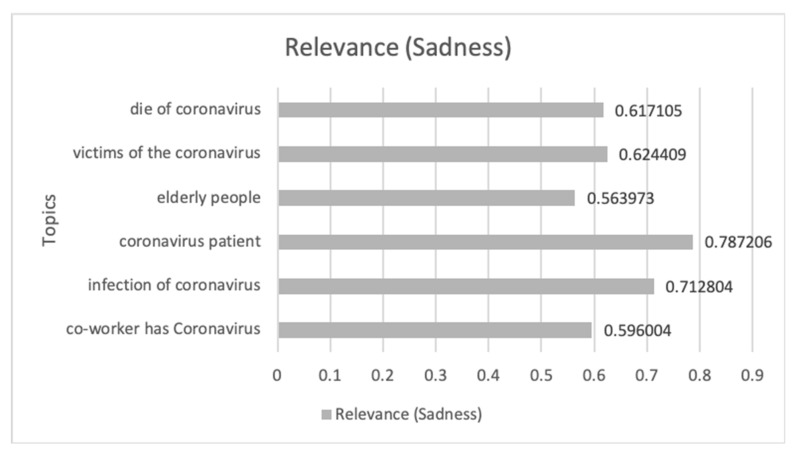
The themes related to COVID-19 and Disgust emotion that have impacted most along with its impact value.

Finally, in Figure 7, the most relevant topics and their impact value on the emotion of disgust are shown. This shows how the management of masks, censorship in the news, and the transmission of the virus in general and especially in groups of elderly people, predominate in this emotion.

#### 3.3.2. Association of Fear Communications Related to Death and Its Emotional Impact during COVID-19 Pandemic

**Figure 8:**
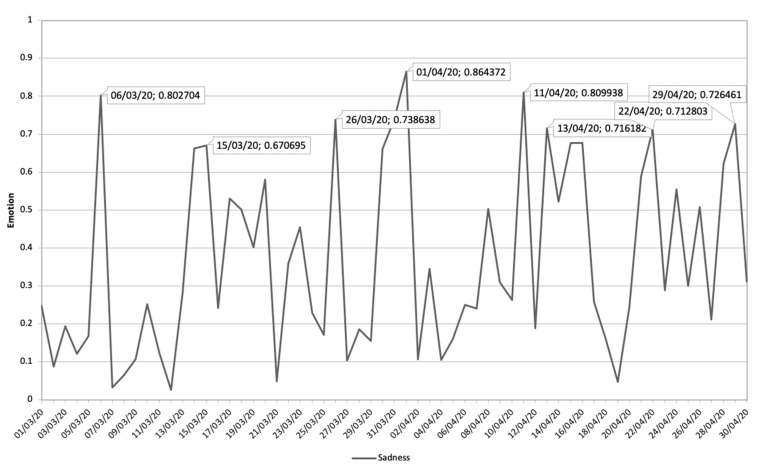
Fear emotion during the period 1 March 2020 until 30 April 2020 related to terms about COVID-19.

Figure 8 shows the evolution of the Fear emotion during the study period, where five peaks can be distinguished, where the emotion shows a sustained decrease over time.

In Table 2, the communications that have had the greatest impact on this temporal progression are analyzed in chronological order from 1 March 2020 to 30 April 2020. Of these communications, the rapid growth of the pandemic in Spain, the overwhelming social security system, and the economic collapse caused by the COVID-19 pandemic are the general themes that have the greatest impact on the emotions addressed. Aspects such as border closures, death forecasts, job losses, defective health material, the Spanish government being overwhelmed, and deaths in residences are the conversations that predominate in digital ecosystems.

**Figure 9:**
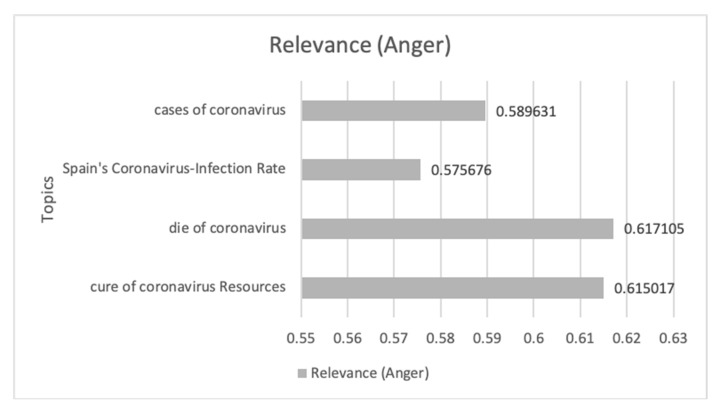
The themes related to COVID-19 and Fear Emotion that have impacted most along with its impact value.

Finally, Figure 9 shows the most relevant issues and their impact value on the emotion Fear. This shows how interest in the state of alarm, the transmission of the virus, emergency health material, and deaths of family members predominate in this emotion.

#### 3.3.3. Association of Anger Communications Related to Lack of Foresight and Its Emotional Impact during COVID-19 Pandemic

**Figure 10:**
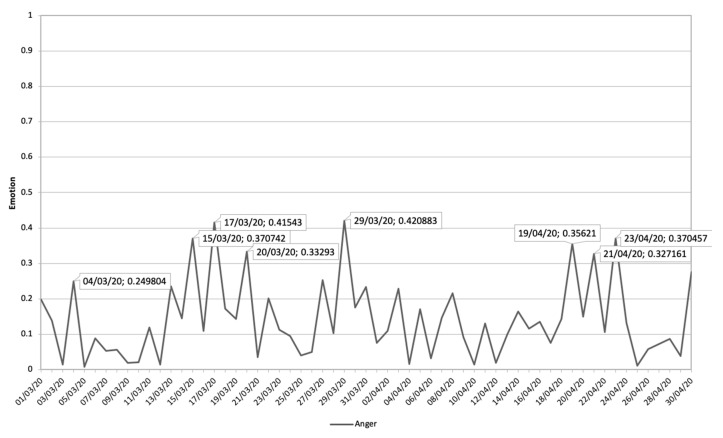
Anger emotion during the period 1 **March** 2020 until 30/04/2020 related to terms about COVID-19.

Figure 10 shows the evolution of the Anger emotion during the study period, where eight peaks can be distinguished where the emotion shows sustained growth over time.

In Table 3, the communications that had the greatest impact on this temporal progression are analyzed in chronological order from 1 March 2020 to 30 April 2020. From these communications, it can be seen that the loss of employment due to lack of foresight, the delay in activating the health alert, and the opacity in the acquisition of health material by the Spanish Government during the crisis by COVID-19 were the driving themes in this case. Aspects such as disinformation for de-escalation, the collapse of the health system, the dubious data on the number of infected and dead people, and the control of the media proposed by members of the Spanish government are some of the conversations that predominate in digital ecosystems.

**Figure 11:**
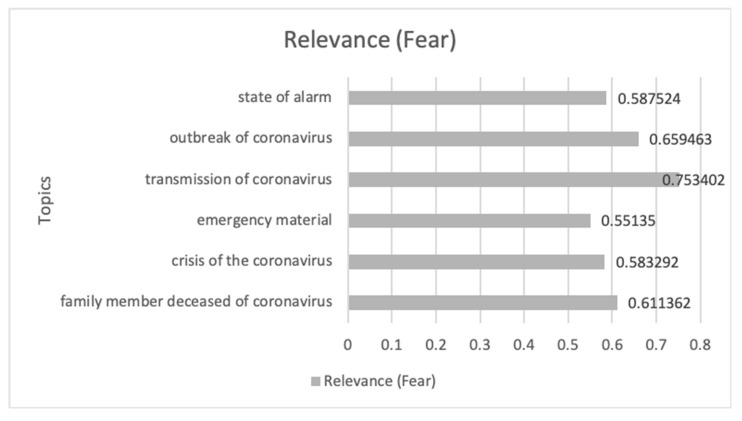
The themes related to COVID-19 and Anger emotion that have impacted most along with its impact value.

Finally, Figure 11 shows the most relevant topics and their impact value on the Anger emotion. This shows how the interest in deaths by a coronavirus, the resources to cure the virus, the diagnosed cases, and the rate of infected, predominate in this emotion. This shows that the lack of prevision predominates in this emotion.

#### 3.3.4. Association of Sadness Communications Related to Safeguarding and Its Emotional Impact during COVID-19 Pandemic

**Figure 12:**
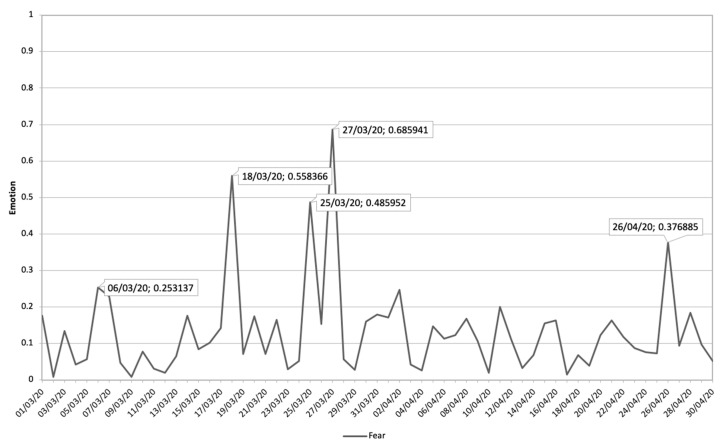
Sadness emotion during the period 1 **March** 2020 until 30 April 2020 related to terms about COVID-19.

Figure 12 shows the evolution of the Sadness emotion during the study period, where eight peaks can be distinguished where the emotion shows sustained growth in time.

In Table 4, the communications that had the greatest impact on this temporal progression are analyzed in chronological order from 1 March 2020 to 30 April 2020. Of these communications, censorship during COVID-19, die of coronavirus, coronavirus patients, infection of coronavirus, and the delay in the incorporation to the labor activity are the general themes that have the greatest impact on the emotion dealt with. Aspects such as political interests, entities that the security of the population due to the virus, sale of necessary material by the COVID-19 pandemic to foreign countries when it is necessary for Spain, healthcare workers exposed to infection by defective health care material are some of the conversations that predominate in digital ecosystems.

**Figure 13:**
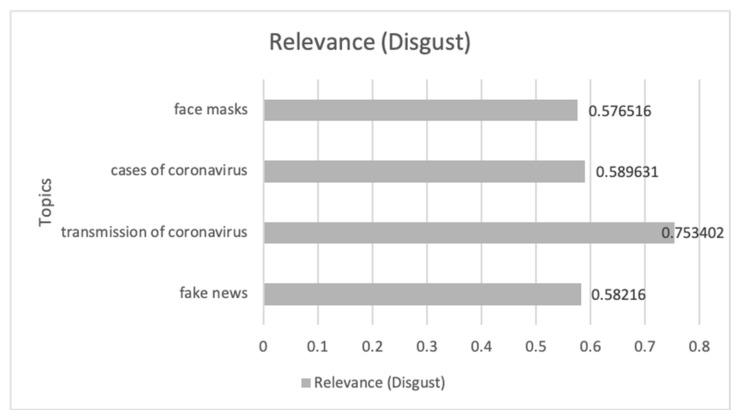
The themes related to COVID-19 and Sadness emotion that have impacted most along with its impact value.

Finally, Figure 13 shows the most relevant topics and their impact value on Sadness’s emotion. This shows how the interest in deaths by COVID-19, patients by COVID-19, the elderly, and infected workers, predominate in this emotion.

### 3.4. Volumes and Flows of Information during the COVID-19 Pandemic

**Figure 14:**
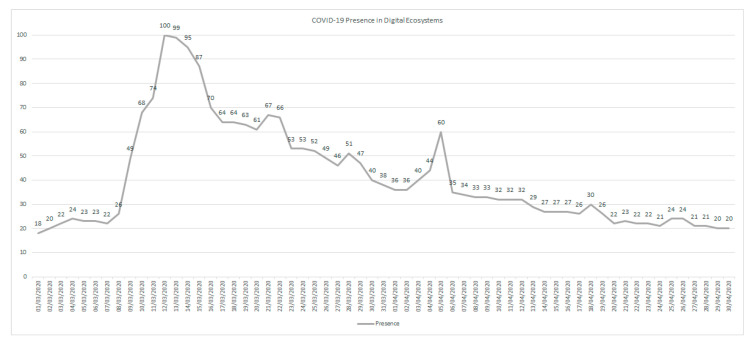
Presence Analysis in Digital Ecosystems during the period 1 March 2020 until 30 April 2020 related to terms about COVID-19.

As shown in Figure 14, the highest presence of the term COVID-19 occurred in the early stages of the pandemic, reaching its highest value on the date when the Government of Spain announced that it would implement the state of alarm and confinement of the population. From that date onwards, there is a downward trend in the use of this term, until 5 April, when an extension of the state of alarm is announced.

During this time, the use of the term COVID-19 followed a decreasing tendency, motivated by the emotions that the population experienced. If at the beginning, the great concern was the virus, the management carried out by the government, the deaths, the social actions, all caused a change in the terms used in the digital ecosystems, with the virus being a secondary problem about the subjects that influence the emotions.

## 4. Conclusions

The spread of pandemics causes uncertainty and fear among the population. This type of crisis, by not adjusting to specific limits, makes risk communication more critical when designing effective strategies [52,53]. Effective risk communication means that all messages can be presented and shared with the population in a transparent, credible, and easily understood communication process. Its main objective is to reduce the knowledge gap between the issuers of information and its recipients to adjust public behavior to proactively address risk [54,55]. The essential elements for reducing risk and avoiding panic among the population are rapid action by public health organizations and truthful and honest information from governments [56].

Even though Rodin et al. [57] indicate that in the case of a crisis in public health, stakeholders are structured in international and national public health organizations, national governments, non-governmental organizations, the media, and citizens, the serious situation experienced in Spain has led to new actors taking on a decisive role in communication, modifying the organizational structure originally designed by the government. Therefore, the little or no dialogue between the government and the social actors that make up the map of the main publics involved in the COVID-19 crisis with different points of view in the face of the pandemic leads to the conclusion that the structure of the stakeholders involved does not determine singular, clear and efficient communication that gives confidence to society.

The analysis of the government’s communication management shows that the messages emitted, mostly with a positive tone, have been offset by a flow of information from other actors in disagreement with government policies. These are mainly channeled by the media and especially the generalist televisions. In Spain, three out of four citizens have used generalist television to keep themselves informed during the pandemic. Television is also the medium most used by Spaniards to seek out different expert opinions. Finally, seven out of ten Spaniards say that the diversity of journalists, approaches, and news items on generalist television help them form their own opinions [58]. This information, sometimes contradictory, that reaches the population makes uncertainty and panic be perceived by the citizens through digital ecosystems.

There are significant differences between the feelings and emotions of the public about COVID-19 analyzed in this study and the tone of the risk communication carried out by the Spanish government and the committee of experts represented in Figure 4.

Risk communication has very close links to the behavioral health issues that affect tens of millions of people. Fear and anxiety about a new disease and what could happen can be overwhelming and cause strong emotions in the population. Through the monitoring of the emotions and the general sentiment of the people across social media about the COVID-19 pandemic reveals that:

Research shows that the current COVID-19 pandemic is creating an added strain on our emotional well-being.

Topics and themes connected to COVID-19 include Management, Social Collaboration, Death, Safeguarding, and Lack of Foresight. Those are strongly related to health and finances, uncertainty about the length of the quarantine, anger over the loss of control, fear of death, illness, loss of employment, economic instability, loss of loved ones, discontent with the Spanish government, transparency, a sense of loneliness and, ultimately, fear of the unknown.

Research results also demonstrate a lot of mixed feelings. It is observed that the same news, information or media communication generated peaks in different emotions, indicating that they are very mixed between sadness, disgust, anger, and fear.

Presence analysis reveals that the term COVID-19 received the highest presence during the early stages of the pandemic, reaching its highest value on the date when the Government of Spain announced that it would implement the state of alarm and confinement of the population. From that date onwards, there is a downward trend in the use of the term COVID-19.

During this time, the use of the term COVID-19 has followed a decreasing tendency, motivated by the emotions that the population has experienced. Initially, as reflected in the study, only the virus (term COVID) was of interest, and later, the consequences and direct impact of the virus on daily life.

## Figures and Tables

**Table 1:** Comments related to Disgust emotion and COVID-19 in digital ecosystems.

Disgust Emotion Related to COVID-19 Comments in Digital Ecosystems	
2 March 2020	The religious community is eager for the Ministry of Health to “point them out” because it leaves them in a “state of defenselessness” and they demand an apology. The evangelicals see it as “very serious” that the Ministry of Health points to a religious group as a possible focus.
23 March 2020	A collapse in funeral homes and mortuaries. Spanish health workers are the worst protected. The government is withholding health materials from autonomous communities. The risk of coronavirus is due to gender roles. Health risk for allowing 8M and then blaming others. Lack of material by the government for testing. Purchase of defective tests by the government. Do not know who sells the defective tests. Ministers choose private health care while criticizing it and asking everyone to go to the public health care system. There are tests for politicians and not for the general population (case of Minister Irene Montero), and they skip the confinement (case of Second Vice-President Pablo Iglesias).
28 March 2020	Purchase of material from the Chinese testing company that sold the failed tests. Announcement of shipment of quantities of masks and the reality is much lower. Fake tests that the government bought when private companies had discovered the deception. Use of false accounts by members of the government to defend the government’s management. Rapid tests do not work.
09 April 2020	Purchase of protection material from companies without guarantee, having lists of companies provided by the EU to buy material with a guarantee. Errors in the death count by the Ministry of Health. Catalonia requests a ban on the army and Guardia Civil hospitals. Spanish Government presumes better management of Europe. Elderly deaths in nursing homes.
10 April 2020	Triumphalist government management of the pandemic.
14 April 2020	Purchase of masks from opaque companies. The government forces companies to provide workers with protective measures when they cannot buy material. The government spends money on protecting cars when there is a lack of material in hospitals and nursing homes.
20 April 2020	Government members fail to comply with confinement. Government management to protect against future malpractice claims. The political use of pandemic management. Use of the Guardia Civil to minimize the anti-government climate.
23 April 2020	Government control of the media. Higher payment for medical equipment by the government. Lack of material for workers and they are forced to return to work. The hiring of companies without guarantees to obtain sanitary material.
28 April 2020	Use of the police and confinement to control complaints from the population. The government admits that it lies about the number of tests performed. False count in the number of deaths.

**Table 2:** Comments related to Fear emotion and COVID-19 in digital ecosystems.

Fear Emotion Related to COVID-19 Comments in Digital Ecosystems	
6 March 2020	Loss of jobs.
18 March 2020	The virus preys on residences. Closing of borders.
25 March 2020	Spain surpasses China in coronavirus deaths with 3434 deaths. The number of deaths continues to rise. The Spanish government now estimates more than three months of economic collapse. Collapsed health system.
27 March 2020	The virus has reached the level of a pandemic. The Spanish government is overwhelmed.
26 April 2020	Censorship by the Spanish Government. Increased unemployment. Defective medical equipment. Increased unemployment. Defective medical equipment.

**Table 3:** Comments related to Anger emotion and COVID-19 in digital ecosystems.

Anger Emotion Related to COVID-19 Comments in Digital Ecosystems	
4 March 2020	Calls to the phone to do the COVID-19 test and they do not attend the patients. Discomfort in the evangelic community when they are pointed out as the focus of COVID-19. Deceased people in Valencia are hiding because of COVID-19.
15 March 2020	The first day of the state of alarm declared by the Spanish Government. Experts warn the government that on Friday the 20th there will be 28,000 infected and 800 dead. Spain exported coronavirus tests until March 15th despite warnings from the WHO. The battle between Pedro Sánchez-Pablo Iglesias is putting the brakes on the anti-epidemic plan.
17 March 2020	More than 100,000 people temporarily lose their jobs. Delay in activating the health alert and border closure with 10,000 cases confirmed by COVID-19.
20 March 2020	Social Security collapsed and lack of medical equipment. Sick people are prioritized according to life expectancy. Loss of jobs. Politicians are skipping confinement, while the population must comply.
29 March 2020	Pandemic announced and no action taken. Purchase of defective medical equipment for companies. Lies about buying from mask suppliers.
19/04/2020	Spain will also be the last in Europe to fall out of favor. Death of old people in retirement homes. Data of deceased/contagious people in doubt. China offered false data of infected and false deaths; more deaths are announced.
21/04/2020	Use of the security forces to control the population and impose censorship Lack of food in old people’s homes.
23/04/2020	The government bought the fake Chinese tests for COVID-19 for more than twice their value. COVID-19’s overcrowding: “Someone’s sleeping on the pillow.” The coronavirus entered Spain on February 15 and by 15 different routes. “De-climbing blindly: Moncloa asks communities for their plan.” Last Monday, the second vice president, Pablo Iglesias, demanded that Sanchez increase his control of the press, and Pedro Sanchez agreed. The government orders the officials to gradually return to work without assuring them of any tests or masks. Pedro Sanchez is in a state of alarm about the previous step to cut benefits for 900,000 civil servants. Political group (PSOE) told parents who wanted to walk their children: “You should have bought a dog”.

**Table 4:** Comments related to Sadness emotion and COVID-19 in digital ecosystems.

Sadness Emotion Related to COVID-19 Comments in Digital Ecosystems	
6 March 2020	Censorship by the Spanish government (Second Vice-President and Minister of Social Rights and Agenda 2030 of the Spanish Government, Pablo Iglesias threatens journalists with prison for reporting). Hidden Coronavirus deaths (Cases from previous months are uncovered). Postponement of economic activity and events due to coronavirus.
15 March 2020	The President of the Spanish Government, Pedro Sánchez, postpones aid to companies and workers. Confinement (State of alarm). Political interests before the security of the population by the virus. Sale of necessary material by the COVID-19 pandemic abroad when it is needed in Spain.
26 March 2020	Government failure to purchase sanitary materials for COVID-19. Death of elderly people in solitude, in homes and residences. The number of coronavirus infections in Spain has risen to 56,188 and there have already been 4089 deaths. “One-third of humanity is already living in the confines of the virus.” The Spanish government admits that it had data that the last week of February was key in the increase of infections in areas such as Madrid and yet it spurred massive events such as 8-M. The tests purchased from China are inaccurate. The companies continue with the wave of dismissals due to the coronavirus.
1 April 2020	Catalonia, to its health care workers in the face of the coronavirus: “Death at home is the best option”. “The EU expects the virus to rebound in weeks”. Government mortgages Spain economically. The Government demands that no one goes on holiday during Easter Week. The rise in deaths. Government with political interests before citizens’ interests.
11 April 2020	Concealment and errors in the deceased/contagious. Death of health care workers. Experts are removed from the management of COVID-19 and replaced by politicians. Castilla-La Mancha sets a record for the number of deaths in a single day.
13 April 2020	The inability of the government to provide protection material to businesses.
22 April 2020	Health workers exposed to contagion by false sanitary material. Use of the Police to censor the population.
29 April 2020	Massive job loss. The health workers are being fired. Defective sanitary equipment. Elderly deaths. False data from the Spanish government regarding COVID-19. Political interests take precedence over health care before and during the pandemic.

## References

[1] Organización Mundial de la Salud (2016). Reglamento Sanitario Internacional.

[2] Regulations E.C. (2020). Statement on the Second Meeting of the International Health Regulations (2005) Emergency Committee Regarding the Outbreak of Novel Coronavirus (2019-nCoV).

[3] JHU CSSE COVID-19 Dashboard by the Center for Systems Science and Engineering (CSSE) at Johns Hopkins University (JHU). https://coronavirus.jhu.edu/map.html.

[4] Arshad Ali S., Baloch M., Ahmed N., Arshad Ali A., Iqbal A. (2020). The outbreak of Coronavirus Disease 2019 (COVID-19)—An emerging global health threat. J. Infect. Public Health.

[5] Yang R., Du G., Duan Z., Du M., Miao X., Tang Y. (2020). Knowledge System Analysis on Emergency Management of Public Health Emergencies. Sustainability.

[6] Maital S., Barzani E. (2020). The Global Economic Impact of COVID-19: A Summary of Research. https://www.neaman.org.il/EN/Files/Global%20Economic%20Impact%20of%20COVID19.pdf.

[7] Bentley J.H. (2012). The Oxford Handbook of World History.

[8] Nazir M., Hussain I., Tian J., Akram S., Mangenda Tshiaba S., Mushtaq S., Shad M.A. (2020). A Multidimensional Model of Public Health Approaches Against COVID-19. Int. J. Environ. Res. Public Health.

[9] Grafton A., Rosenberg D. (2010). Cartographies of Time: A History of the Timeline.

[10] Guidry J.P.D., Jin Y., Orr C.A., Messner M., Meganck S. (2017). Ebola on Instagram and Twitter: How health organizations address the health crisis in their social media engagement. Public Relat. Rev..

[11] James J. (1999). Globalization, Information Technology and Development.

[12] Baldwin R. (2016). The Great Convergence: Information Technology and the New Globalization.

[13] Chou W.S., Hunt Y.M., Beckjord E.B., Moser R.P., Hesse B.W. (2009). Social Media Use in the United States: Implications for Health Communication. J. Med. Internet Res..

[14] Gesser-Edelsburg A., Shir-Raz Y., Hayek S., Sassoni-Bar Lev O. (2015). What does the public know about Ebola? The public’s risk perceptions regarding the current Ebola outbreak in an as-yet unaffected country. Am. J. Infect. Control.

[15] Palenchar M.J., Heath R.L. (2007). Strategic risk communication: Adding value to society. Public Relat. Rev..

[16] McKie D., Heath R.L. (2016). Public relations as a strategic intelligence for the 21st century: Contexts, controversies, and challenges. Public Relat. Rev..

[17] Conrow E.H., Pohlmann L.D. (2004). Effective Risk Management: Some Keys to Success, Second Edition. Insight.

[18] Ruiz de Azua S., Ozamiz-Etxebarria N., Ortiz-Jauregui M.A., Gonzalez-Pinto A. (2020). Communicative and Social Skills among Medical Students in Spain: A Descriptive Analysis. Int. J. Environ. Res. Public Health.

[19] Covello V.T. (2001). Risk communication, the West Nile virus epidemic, and bioterrorism: Responding to the communication challenges posed by the intentional or unintentional release of a pathogen in an urban setting. J. Urban Health Bull. N. Y. Acad. Med..

[20] Arrow K.J. (1951). Social Choice and Individual Values.

[21] Ministerio de la Presidencia (2020). Real Decreto 463/2020, de 14 de Marzo, por el que se Declara el Estado de Alarma para la Gestión de la Situación de Crisis Sanitaria Ocasionada por el COVID-19.

[22] Centro de Coordinación de Alertas y Emergencias Sanitarias, Ministerio de Sanidad, Gobierno de España Actualización no 123. Enfermedad por el Coronavirus (COVID-19). https://www.mscbs.gob.es/en/profesionales/saludPublica/ccayes/alertasActual/nCov-China/documentos/Actualizacion_123_COVID-19.pdf.

[23] Ministry of Health (2020). Evolution of Reported Cases, Deaths and Recovered Cases from the COVID-19 Pandemic in Spain.

[24] Data E.P. Masks that the Spanish Government Has Distributed to Each Autonomous Community from March 10 to May 29. https://www.epdata.es.

[25] Graham M.W. (2014). Government communication in the digital age: Social media’s effect on local government public relations. Public Relat. Inq..

[26] Instituto Nacional de Estadística (2019). Cifras de Población (CP) a 1 de Julio de 2019.

[27] Jose T., Babu S.S. (2019). Detecting spammers on social network through clustering technique. J. Ambient Intell. Humaniz. Comput..

[28] Zheng X., Zeng Z., Chen Z., Yu Y., Rong C. (2015). Detecting spammers on social networks. Neurocomputing.

[29] Hoyt R.E., Snider D., Thompson C., Mantravadi S. (2016). IBM Watson Analytics: Automating Visualization, Descriptive, and Predictive Statistics. JMIR Public Health Surveill..

[30] Cao X., MacNaughton P., Deng Z., Yin J., Zhang X., Allen J. (2018). Using Twitter to Better Understand the Spatiotemporal Patterns of Public Sentiment: A Case Study in Massachusetts, USA. Int. J. Environ. Res. Public Health.

[31] Guidi G., Miniati R., Mazzola M., Iadanza E. (2016). Case Study: IBM Watson Analytics Cloud Platform as Analytics-as-a-Service System for Heart Failure Early Detection. Futur. Internet.

[32] Palomino M., Taylor T., Göker A., Isaacs J., Warber S. (2016). The Online Dissemination of Nature–Health Concepts: Lessons from Sentiment Analysis of Social Media Relating to “Nature-Deficit Disorder”. Int. J. Environ. Res. Public Health.

[33] Al Marouf A., Hossain R., Kabir Rasel Sarker M.R., Pandey B., Tanvir Siddiquee S.M. (2019). Recognizing Language and Emotional Tone from Music Lyrics using IBM Watson Tone Analyzer. Proceedings of the 2019 IEEE International Conference on Electrical, Computer and Communication Technologies (ICECCT).

[34] Brin S., Page L. (1998). The anatomy of a large-scale hypertextual Web search engine. Comput. Netw. ISDN Syst..

[35] Kant N., Puri R., Yakovenko N., Catanzaro B. (2018). Practical Text Classification With Large Pre-Trained Language Models. arXiv.

[36] Peláez J.I., Martínez E.A., Vargas L.G. (2019). Decision making in social media with consistent data. Knowl.-Based Syst..

[37] Peláez J.I., Cabrera F.E., Vargas L.G. (2018). Estimating the importance of consumer purchasing criteria in digital ecosystems. Knowl.-Based Syst..

[38] Peláez J.I., Martínez E.A., Vargas L.G. (2019). Products and services valuation through unsolicited information from social media. Soft Comput..

[39] Pelaez J.I., Martinez E.A., Vargas L.G. (2018). Consistency in Positive Reciprocal Matrices: An Improvement in Measurement Methods. IEEE Access.

[40] Bird S., Klein E., Loper E. (2009). Natural Language Processing with Python.

[41] Moreno A., Redondo T. (2016). Text Analytics: The convergence of Big Data and Artificial Intelligence. Int. J. Interact. Multimed. Artif. Intell..

[42] Singh P.K., Shahid Husain M. (2014). Methodological Study Of Opinion Mining And Sentiment Analysis Techniques. Int. J. Soft Comput..

[43] van der Meer T.G.L.A. (2016). Automated content analysis and crisis communication research. Public Relat. Rev..

[44] Krippendorff K. (2004). Content Analysis: An Introduction to Its Methodology.

[45] de las Heras-Pedrosa C., Jambrino-Maldonado C., Iglesias-Sánchez P.P., Millán-Celis E. (2020). Populism and Independence Movements in Europe: The Catalan-Spanish Case. Soc. Sci..

[46] Secretaria General de Sanidad (2020). Actualización n°13. Numonía por Nuevo Conavirus (2019-nCov) en Wuhan, Provincia de Hubei, (China).

[47] Jefatura del Estado (2020). Real Decreto-ley 10/2020, de 29 de Marzo, por el que se Regula un Permiso Retribuido Recuperable Para las Personas Trabajadoras por Cuenta Ajena que no Presten Servicios Esenciales, con el fin de Reducir la Movilidad de la Población en el Contexto de la l.

[48] Jefatura del Estado (2020). Real Decreto-ley 8/2020, de 17 de Marzo, de Medidas Urgentes Extraordinarias Para Hacer Frente al Impacto Económico y Social del COVID-19.

[49] Diario Expansión (2020). El Número de Trabajadores Afectados por ERTE se Aproxima ya a los dos Millones bajo 374.150 Expedientes. https://www.expansion.com/economia/2020/04/03/5e87329ae5fdea2d618b45ae.html.

[50] Europa Press (2020). Marzo se Convierte en el mes de Mayor Consumo de TV en España Desde que hay Registros, Según un Estudio. https://www.europapress.es/sociedad/noticia-marzo-convierte-mes-mayor-consumo-tv-espana-hay-registros-estudio-20200331144158.html#:~:text=El%20mes%20de%20marzo%20de,de%20la%20pandemia%20del%20coronavirus.

[51] Europa Press (2020). Abril Marca un Récord Histórico Mensual de Consumo Televisivo: 5 Horas y 2 Minutos Diarios por Persona. https://www.europapress.es/sociedad/noticia-abril-marca-record-historico-mensual-consumo-televisivo-horas-minutos-diarios-persona-20200501122200.html#:~:text=mayo%20de%202020-,Abril%20marca%20un%20r%C3%A9cord%20hist%C3%B3rico%20mensual%20de%20consumo%20televisivo%3A%205,2%20minutos%20diarios%20por%20persona&text=Respecto%20a%20la%20cobertura%20televisiva,de%20la%20poblaci%C3%B3n%20de%20Espa%C3%B1a.

[52] Kenis P., Schol L.G.C., Kraaij-Dirkzwager M.M., Timen A. (2019). Appropriate Governance Responses to Infectious Disease Threats: Developing Working Hypotheses. Risk Hazards Cris. Public Policy.

[53] Campbell-Lendrum D., Manga L., Bagayoko M., Sommerfeld J. (2015). Climate change and vector-borne diseases: What are the implications for public health research and policy?. Philos. Trans. R. Soc. B Biol. Sci..

[54] Frewer L. (2004). The public and effective risk communication. Toxicol. Lett..

[55] Arvai J., Rivers L. (2014). Effective Risk Communication.

[56] Sellnow T.L., Ulmer R.R., Seeger M.W., Littlefield R.S. (2009). Effective Risk Communication.

[57] Rodin P., Ghersetti M., Odén T. (2018). Disentangling rhetorical subarenas of public health crisis communication: A study of the 2014–2015 Ebola outbreak in the news media and social media in Sweden. J. Conting. Cris. Manag..

[58] UTECA I Barómetro Sobre la Percepción Social de la Televisión en Abierto. https://uteca.tv/i-barometro-tv-en-abierto/.

